# Cognitive and emotional mechanisms of psychological resilience in post-disaster contexts: the influence of risk perception, prior experience, and cognitive resilience in a processual explanatory model

**DOI:** 10.3389/fsoc.2026.1761001

**Published:** 2026-02-17

**Authors:** Mariana Floricica Călin, Mihaela Luminița Sandu, Silvia Raluca Matei

**Affiliations:** Faculty of Psychology and Educational Sciences, Ovidius University, Constanta, Romania

**Keywords:** cognitive resilience, conditional process analysis, disaster context, moderated mediation, prior disaster experience, psychological resilience, risk perception

## Abstract

**Introduction:**

In the context of the increasing frequency and intensity of climate-related natural disasters, identifying the psychological mechanisms that support adaptive functioning has become a critical research priority. Psychological resilience is conceptualized as a dynamic, context-dependent adaptive process shaped by cognitive appraisals of risk, regulatory cognitive mechanisms, and prior disaster experience. The present study examined the relationship between risk perception and psychological resilience by testing the mediating role of cognitive resilience and the conditional influence of prior disaster experience and hazard type.

**Methods:**

The sample consisted of 172 higher-education teachers from Romania, predominantly from the Dobrogea region. Data were collected using the psychometrically validated Resilience to Natural Disasters Questionnaire (α = 0.79–0.89). Conditional process analyses were conducted in SPSS (v.28) using PROCESS Models 1, 4, and 7, with 5,000 bootstrap resamples, to test mediation, moderation, and moderated mediation effects.

**Results:**

Cognitive resilience significantly mediated the relationship between risk perception and psychological resilience, with the magnitude of the indirect effect varying as a function of hazard type. The indirect effect of prior disaster experience through cognitive resilience remained consistent across hazard contexts. Cognitive mediation was stronger in contexts characterized by higher unpredictability (fires) than in more controllable situations (floods). Heightened cognitive activation was associated with lower levels of psychological resilience, suggesting a compensatory mechanism under conditions of elevated threat. Prior disaster experience further shaped the strength of these associations across models.

**Discussion:**

The findings support a processual and context-sensitive model of psychological resilience, highlighting the dynamic interplay between risk perception, cognitive regulation, experiential factors, and situational characteristics. These results provide an empirical foundation for differentiated post-disaster psychological interventions tailored to individuals' cognitive and experiential profiles.

## 1 Introduction

In recent decades, psychological resilience has become a central construct in health and trauma psychology, being conceptualized as a dynamic process of positive adaptation in the face of adversity (Bonanno, 2021). In the context of natural disasters, resilience reflects the capacity of individuals and communities to manage intense stressors, restore emotional balance, and re-establish psychosocial functioning following disruptive events (Norris et al., 2008). Extreme weather phenomena—such as floods, fires, earthquakes, and hurricanes—constitute not only physical threats but also complex psychological challenges that require the mobilization of cognitive, emotional, and behavioral regulatory mechanisms (Brooks et al., 2019; Crane et al., 2022; Morganstein and Ursano, 2020; American Counseling Association, 2025).

The increasing frequency and intensity of these extreme events, largely attributed to global climate change (Intergovernmental Panel on Climate Change, 2023), underscore the need for psychological research on mechanisms supporting effective adaptation and post-disaster emotional recovery.

Contemporary research emphasizes that resilience in crisis contexts is not a stable trait but a flexible and context-sensitive process shaped by the interaction of personal factors (e.g., risk perception, cognitive resources, prior experience), social resources (e.g., community support), and institutional structures (e.g., preparedness policies and intervention infrastructures) (Kalisch et al., 2019; Chen et al., 2024). This perspective aligns with ecological and multisystemic models of resilience, which conceptualize adaptation as emerging from dynamic interdependencies between individual, cognitive, and systemic levels (Ungar, 2021).

A key component of adaptive functioning in disaster contexts is risk perception, defined as the set of cognitive and affective representations through which individuals evaluate the probability and severity of threatening events (Slovic, 2016; Wachinger et al., 2013). Risk perception plays a central role in shaping both preventive behaviors and emotional responses, such that balanced and realistic appraisals facilitate functional coping, whereas risk overestimation or denial may lead to maladaptive outcomes (Akter and Khanal, 2020; Hori and Shaw, 2014). In the context of natural hazards, risk perception is closely linked to levels of preparedness and is strongly influenced by prior exposure to similar events (Paton, 2019).

Within this framework, cognitive resilience represents a critical regulatory mechanism. Cognitive resilience refers to the capacity to employ higher-order cognitive processes—such as planning, cognitive flexibility, and positive reappraisal—to manage uncertainty and stress (Tugade and Fredrickson, 2004). Empirical evidence indicates that cognitive resilience mediates the relationship between perceived risk and emotional adjustment, contributing to psychological stability and recovery following adverse events (Feder et al., 2019; Broche-Pérez et al., 2025; Chen et al., 2023; Xu et al., 2025). Individuals who are able to cognitively reinterpret threatening experiences in an adaptive manner tend to display higher levels of psychological resilience and more efficient emotional regulation.

From a methodological perspective, conditional process models of mediation and moderation provide a robust framework for examining how prior disaster experience influences the relationships between risk perception, cognitive adaptation, and psychological resilience (Hayes, 2022). Recent studies suggest that direct exposure to natural disasters does not exert a uniform effect on resilience outcomes; rather, it may either strengthen adaptive mechanisms or increase vulnerability, depending on event characteristics, perceived controllability, and available cognitive resources (Bano et al., 2025). For example, educators previously exposed to hurricanes and severe storms have been shown to develop heightened risk awareness and transformed resilience grounded in cognitive reflection and meaning-making processes (Bano et al., 2025). Similarly, research on community-level adaptation highlights the role of collective learning and social capital in shaping post-disaster resilience trajectories (Partelow, 2021).

At the individual level, resilience is further facilitated by meaning-making processes that enable individuals to transform adverse experiences into opportunities for growth, as described by benefit-finding and posttraumatic growth frameworks (Tugade and Fredrickson, 2004; Tedeschi et al., 2018). Complementarily, the adaptive calibration model posits that cognitive and emotional responses to stress are calibrated according to the intensity and unpredictability of the threat, explaining why highly uncertain contexts—such as fires—demand more complex cognitive engagement than comparatively predictable events like floods (Del Giudice et al., 2011, 2018).

Building on these theoretical perspectives, the present study investigates the relationships between risk perception, cognitive resilience, and psychological resilience in the context of natural disasters, with particular attention to the moderating role of prior disaster experience and hazard type. Using conditional process analysis (Hayes, 2022), the study examines both direct and indirect pathways through which risk perception influences psychological resilience, as well as the contextual conditions under which these effects vary. By adopting a processual and context-sensitive approach, the research aims to advance understanding of resilience as a multidimensional adaptive process and to inform the development of differentiated psychological interventions tailored to the cognitive and experiential profiles of disaster-exposed populations.

Within this framework, psychological resilience is conceptualized as a dynamic adaptive outcome reflecting individuals' capacity to maintain or restore emotional balance following exposure to natural disasters (Bonanno, 2021; Norris et al., 2008) and is operationalized as the primary outcome variable (Y). Cognitive resilience is defined as a set of higher-order cognitive regulatory processes, including cognitive flexibility, planning, and positive reappraisal (Tugade and Fredrickson, 2004; Feder et al., 2019), and is measured as a distinct subcomponent of individual resilience functioning as a mediating mechanism (M). Risk perception refers to individuals' cognitive and affective appraisals of disaster-related threats (Slovic, 2016; Wachinger et al., 2013) and is operationalized through context-specific items assessing perceived severity and concern, serving as a key predictor variable (X).

Conceptually, cognitive resilience is treated as a regulatory process operating during exposure to threat, whereas psychological resilience represents an adaptive outcome reflecting post-exposure emotional balance and functional adjustment, thereby preventing conceptual overlap between mediator and outcome variables.

Prior disaster experience is conceptualized as direct exposure to at least one natural disaster event and is operationalized as a dichotomous variable (with vs. without experience), acting either as an independent predictor or as a moderator (W), depending on the analytical model. This explicit alignment between conceptual definitions, measurement strategies, and analytical roles ensures internal coherence and supports the interpretation of the conditional process analyses.

**Figure 1:**
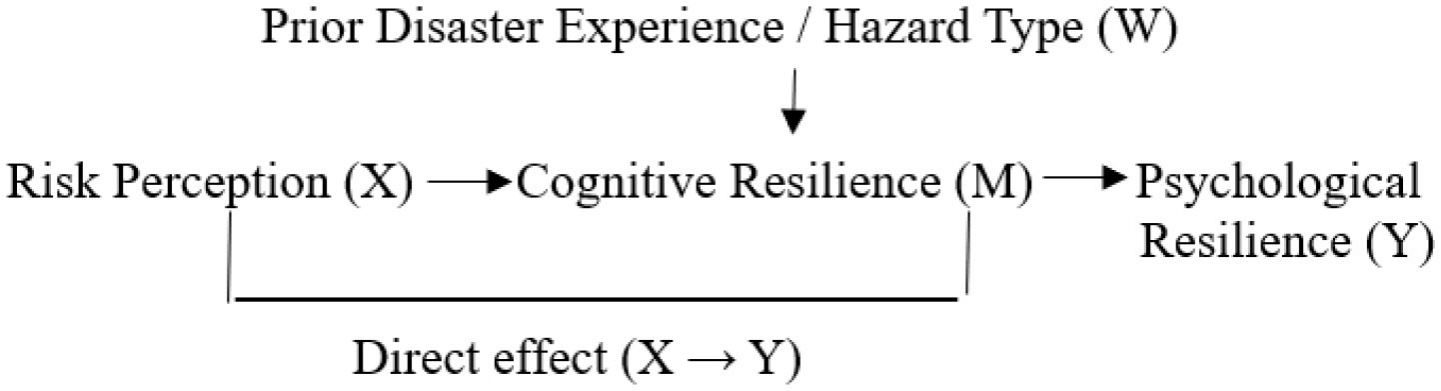
Proposed conditional process model.

The proposed conceptual framework and hypothesized direct, indirect, and conditional relationships among the study variables are summarized in Figure 1.

Risk perception (X) is hypothesized to influence psychological resilience (Y) both directly and indirectly through cognitive resilience (M). Prior disaster experience and hazard type (W) are specified as contextual moderators of the indirect and selected direct pathways, in line with the conditional process framework (Hayes, 2022).

## 2 Materials and methods

### 2.1 Participants

The research sample consisted of 172 higher education teachers from Romania. Participants were recruited using non-probabilistic sampling strategies, specifically convenience sampling and snowball sampling.

The target population of the present study consists of higher education teachers working in academic institutions located in regions exposed to recurrent natural hazards. This professional group was selected due to its relatively homogeneous educational background, high cognitive demands, and institutional responsibilities related to risk awareness, information dissemination, and adaptive functioning in crisis contexts. Focusing on higher education teachers allows for the examination of cognitive and psychological resilience mechanisms within a population characterized by advanced cognitive resources and structured professional roles.

Given the non-probabilistic sampling strategy employed, the findings are not intended to be generalized to the broader population or to all occupational groups. Instead, the results are analytically generalizable to similar professional populations with comparable cognitive profiles and exposure contexts. This approach supports theory-driven inference regarding psychological mechanisms of resilience, while acknowledging limitations in statistical representativeness and external validity.

Non-probabilistic sampling strategies, including convenience and snowball sampling, are widely used in exploratory and applied psychological research when access to a clearly delimited population is limited and the primary research objective is to examine underlying psychological mechanisms rather than to generate population-level prevalence estimates (Etikan et al., 2015; Bryman, 2016; Creswell and Plano Clark, 2018). Such approaches are considered methodologically appropriate for theory-driven studies focusing on mediation and moderation processes within relatively homogeneous professional groups, where analytical generalization and the investigation of process-level relationships are prioritized over statistical representativeness.

Convenience sampling allowed the inclusion of participants who were readily accessible and willing to take part in the study, while snowball sampling facilitated the recruitment of additional respondents belonging to the same professional population. This combined strategy ensured adequate sample size and internal coherence, while maintaining relevance to the targeted cognitive and experiential profile. Although non-probabilistic sampling limits statistical generalizability, it is considered appropriate for theory-driven studies investigating mediation and moderation processes within relatively homogeneous groups (Hayes, 2022; Pallant, 2020).

### 2.2 Measures

For evaluating the constructs included in the research hypotheses, the *Resilience to Natural Disasters Questionnaire* was used, designed to assess risk perception and individual resilience in disaster contexts, specifically fires and floods. The questionnaire comprises two context-specific versions (fire and flood) that share an identical structural framework, while the formulation of the items has been tailored to the specific context of each hazard. The number of items and response formats are identical across versions, ensuring measurement comparability.

Risk perception was measured using 10–11 items that captured awareness, cognitive and emotional assessments, and preventive behaviors, with a 5-step Likert scale (1 = “never”, 5 = “always”). Individual resilience was assessed through four components: psychological, cognitive, adaptive, and institutional, each measured by four items on the same scale. In addition, procedural knowledge related to fire or flood response was assessed using 10 dichotomous items (yes/no), reflecting familiarity with hazard-specific operational steps.

Exploratory factor analysis (EFA) (Table 1) confirmed a consistent and reliable factor structure for all six scales of the instrument, with KMO values ranging from 0.720 to 0.813 and significant Bartlett tests (*p* < 0.001), indicating the adequacy of the data for dimensional reduction.

**Table 1:** Results of exploratory factor analysis and internal consistency for the scales included in the questionnaire on behaviors and perceptions in emergencies (fires and floods).

**Scale**	**SME**	**Bartlett χ^2^(df)**	**No. of extracted factors**	**Variance explained (cumulative)**	**Factorial loads**	**Internal consistency (Cronbach α)**
Knowledge of fire response procedures	0.813	772.824^***^ (55)	2	60.75%	0.45–0.93	0.899
Knowledge of flood response procedures	0.787	782.287^***^ (28)	1	49.56%	0.50–0.90	0.885
Perception of the risk associated with fires	0.746	230.979^***^ (45)	3	60.85%	0.29–0.84	0.791
Flood risk perception	0.720	483.474^***^ (45)	2	42.29%	0.44–0.80	0.871
Individual fire resilience	0.802	865.432^***^ (120)	4	62.30%	0.40–0.81	0.857
Individual flood resilience	0.789	842.617^***^ (120)	4	60.84%	0.42–0.79	0.861

The asterisks indicate levels of statistical significance (^*^p < 0.05, ^**^p < 0.01, ^***^p < 0.001).

The number of factors extracted ranged from one to four, reflecting the theoretical complexity specific to each construct. The total variance explained, ranging from 42.29% to 62.30%, falls within the accepted psychometric limits for multidimensional instruments. Cronbach's α coefficient values, ranging from 0.791 to 0.899, indicate high internal consistency and robust conceptual stability for each scale. Overall, these results support the construct validity and internal reliability of the instrument for research on risk perception, procedural knowledge, and psychological resilience in emergency contexts (Field, 2024).

Following these psychometric evaluations, the instrument was considered appropriate for application in the main stage of the study, with the corresponding results presented in the next section.

### 2.3 Procedure

The data collection was conducted online by distributing the „Resilience to natural disasters„ questionnaire in electronic format to teachers in higher education institutions. Participants were informed about the overall purpose of the study, the anonymity and confidentiality of their responses, and were asked to express informed consent before completing the questionnaire.

The average completion time was about 15–20 min. The answers were collected in a single session, with no time limit imposed, and participants were free to pause at any time. No material rewards were offered for participation.

The data was centralized and coded in SPSS (version 28) (BM Corp. IBM SPSS Statistics for Windows (Version 28.0), 2025), being used exclusively for scientific purposes.

To test the proposed hypotheses, a series of conditional process models were specified using the PROCESS macro for SPSS (v4.2; Hayes, 2022). PROCESS Model 4 was employed to examine simple mediation effects, testing whether cognitive resilience functions as an explanatory mechanism linking predictor variables to psychological resilience. PROCESS Model 7 was used to test moderated mediation, allowing the assessment of whether the indirect effects of risk perception vary as a function of contextual moderators, namely prior disaster experience and hazard type. In addition, PROCESS Model 1 was applied to examine simple moderation effects, testing whether prior disaster experience conditions the direct relationship between risk perception and adaptive resilience. Indirect effects were estimated using bias-corrected bootstrapping procedures with 5,000 resamples, providing robust confidence intervals. Together, these models provide an integrated analytical framework suitable for theory-driven research that simultaneously examines direct, indirect, and conditional effects.

## 3 Results

### 3.1 Hypothesis testing

**Hypothesis 1 (H1)**. The effect of previous experience (X) on psychological resilience (Y) is transmitted indirectly through cognitive resilience (M), and the intensity of this indirect effect varies depending on the type of hazard (W: flood vs. fire). In other words, the mechanism of mediation through cognitive resilience is contingent upon the nature of the emergency, indicating a moderate mediation effect.

The hypothesis assumes a moderated mediation model (PROCESS Model 7), in which previous experience (X, coded 0 = without, 1 = with) influences psychological resilience (Y) through cognitive resilience (M), and this indirect effect is examined as a function of hazard type (W: 0 = fire, 1 = flood). Accordingly, a moderated mediation analysis (Model 7; Hayes, 2018) was conducted to test the mediating role of cognitive resilience in the relationship between prior disaster experience and psychological resilience, while assessing whether this indirect effect varies across hazard contexts (fire vs. flood) (Table 2).

**Table 2:** Results of the moderate mediation analysis (PROCESS Model 7, *N* = 172).

**Effect**	**b**	**Standard error (SE)**	***t***	***p***	**95% CI (LL, UL)**
**Model on M (ReCog2)**					
Experience (X)	0.721	0.122	5.892	0.000	(0.480, 0.963)
Hazard (W)	3.047	0.272	11.204	0.000	(2.510, 3.583)
Experience × Hazard	−0.064	0.245	−0.261	0.794	(−0.547, 0.419)
**Model pe Y (RePsih2)**					
Experience (X)	0.814	0.153	5.318	0.000	(0.512, 1.117)
ReCog2 (M)	−0.156	0.069	−2.264	0.025	(−0.293, −0.020)
**Indirect effects X**→**M**→**Y**					
Hazard = 0 (fire)	−0.118	0.065	—	—	(−0.253, −0.001)
Hazard = 1 (flood)	−0.108	0.070	—	—	(−0.270, 0.001)
**Index of moderate mediation**	0.010	0.047	—	—	(−0.093, 0.110)

The results show that previous experience significantly predicts cognitive resilience [b = 0.721, SE = 0.122, *t* = 5.892, *p* < 0.001, 95% CI (0.480, 0.963)], indicating that individuals with prior disaster exposure tend to mobilize higher levels of cognitive resources for emergency management. In addition, hazard type has a significant direct effect on cognitive resilience (b = 3.047, *p* < 0.001), suggesting that flood-related scenarios are associated with stronger cognitive activation compared to fire-related contexts. However, the interaction between experience and hazard type is not statistically significant (b = −0.064, *p* = 0.794), indicating that the relationship between prior experience and cognitive resilience does not vary as a function of hazard type.

On the outcome pathway (Y), previous experience exhibits a robust positive direct effect on psychological resilience (b = 0.814, *p* < 0.001), confirming its protective role. Cognitive resilience, in turn, shows a significant negative association with psychological resilience (b = −0.156, *p* = 0.025), suggesting that heightened cognitive engagement in coping strategies may be accompanied by a temporary reduction in perceived emotional resources, potentially reflecting cognitive overload or excessive preoccupation under stress.

Analysis of conditional indirect effects indicates that prior experience influences psychological resilience through cognitive resilience in both the fire scenario [b = −0.118, 95% CI (−0.253, −0.001)] and the flood scenario [b = −0.108, 95% CI (−0.270, 0.001)]. However, the index of moderated mediation is not statistically significant [Index = 0.010, 95% CI (−0.093, 0.110)], indicating that the magnitude of the indirect effect does not differ significantly across hazard types.

Taken together, these findings support the mediating role of cognitive resilience in the relationship between prior disaster experience and psychological resilience, while providing no empirical evidence that this mediation mechanism is contingent upon hazard type.

**Hypothesis 2 (H2)**. The relationship between risk perception (X) and psychological resilience (Y) is mediated by cognitive resilience (M), and this mediation path is moderated by the type of emergency (W: floods,/fire).

The detailed results of the moderate mediation analysis are summarized in Tables 3–5, and the integrated interpretation of the model is presented below.

**Table 3:** Moderate mediation analysis results (PROCESS model 7, *N* = 172, 5,000 bootstrap samples).

**Predictor**	**B**	**Standard error (SE)**	***t***	***p***	**95% CI LL**	**95% CI UL**
**Mediator (ReCog2)**						
Constant	4.6744	0.111	42.03	<0.001	4.455	4.894
Risk perception (X)	0.0994	0.010	10.17	<0.001	0.080	0.119
Situation (W)	3.0465	0.222	13.70	<0.001	2.607	3.486
X × W	0.1097	0.020	5.61	<0.001	0.071	0.148
**Addictive (RePsih2)**						
Constant	6.7300	0.269	25.03	<0.001	6.199	7.261
Risk perception (X)	0.1687	0.011	14.97	<0.001	0.146	0.191
Cognitive resilience (M)	−0.3950	0.052	−7.58	<0.001	−0.498	−0.292

**Table 4:** Conditional indirect effects of risk perception on psychological resilience through cognitive resilience at different moderator levels (W).

**Moderator (W)**	**Indirect effect**	**Boot SE**	**Boot 95% CI LL**	**Boot 95% CI UL**
Floods (0)	−0.0176	0.0067	−0.0313	−0.0050
Fire (1)	−0.0609	0.0095	−0.0810	−0.0435

**Table 5:** Moderate mediation index.

**Presenter**	**Index**	**Boot SE**	**Boot 95% CI LL**	**Boot 95% CI UL**
W	−0.0433	0.0100	−0.0651	−0.0254

Moderate Mediation Analysis—PROCESS Model 7 (Hayes, 2022) (Table 3) confirms hypothesis H2, which posits that the relationship between risk perception (X) and psychological resilience (Y) is mediated by cognitive resilience (M), and the intensity of this mediation pathway is contingent upon the type of emergency (W).

The results show that risk perception has a significant positive effect on cognitive resilience [B = 0.0994, SE = 0.010, *t* = 10.17, *p* < 0.001, 95% CI (0.080, 0.119)], suggesting that a more acute perception of risk activates cognitive coping mechanisms such as planning, anticipating, and rationalizing the crisis. Also, the significant interaction between risk perception and the type of situation (X × W) [B = 0.1097, SE = 0.020, *t* = 5.61, *p* < 0.001, 95% CI (0.071, 0.148)] indicates that the relationship between risk perception and cognitive resilience is influenced by the nature of the threatening event, being stronger in conditions perceived as severe or unpredictable (such as fires).

In the final model, risk perception has a significant positive effect on psychological resilience [B = 0.1687, SE = 0.011, *t* = 14.97, *p* < 0.001, 95% CI (0.146, 0.191)], while cognitive resilience has a significant adverse effect on the same variable [B = −0.3950, SE = 0.052, *t* = −7.58, *p* < 0.001, 95% CI (−0.498, −0.292)]. This combination suggests the existence of a compensatory mechanism between the cognitive and affective components of resilience, in which an overactivation of cognitive control can temporarily diminish the emotional resources involved in psychological adaptation.

The results presented in Table 4 indicate that both indirect effects are negative and statistically significant, with their magnitude varying depending on the type of event. In the case of floods (W = 0), the indirect effect is −0.0176 [Boot SE = 0.0067, 95% CI: (−0.0313, −0.0050)], indicating a weak but consistent mediation of cognitive resilience between risk perception and psychological resilience. This suggests that in more predictable and controllable situations, the cognitive component of resilience plays a moderating role, facilitating risk processing without significantly affecting overall psychological balance.

In contrast, in the case of fires (W = 1), the indirect effect is −0.0609 [Boot SE = 0.0095, 95% CI (−0.0810, −0.0435)], of much higher magnitude, indicating an intensified cognitive mediation. This result highlights the fact that, in situations perceived as more severe, unpredictable, and with a low degree of personal control, individuals activate more complex cognitive resources to manage stress and uncertainty, albeit at a temporary higher psychological cost, as reflected in the decrease in the general level of psychological resilience.

The clear difference between the two conditions validates the existence of a mediation conditioned by the situational context, confirming that cognitive resilience more strongly mediates the relationship between risk perception and psychological resilience in situations perceived as threatening and unstable. This result is aligned with recent studies on contextual models of resilience (Bonanno, 2021; Kalisch et al., 2019; Chen et al., 2024) who claim that cognitive adaptation processes are more active and demanding under conditions of acute stress, influencing emotional balance and overall psychological performance.

In theoretical terms, this result suggests that cognitive resilience functions as a compensatory mechanism, allowing functionality to be maintained in the face of extreme threats, but can result in a temporary decrease in affective resources. On the other hand, in more controllable situations, cognitive involvement is effective and proportionate, contributing to a balanced adaptation.

Therefore, the analysis confirms the existence of a robust conditional indirect effect, which validates the moderate mediation model proposed in the H2 hypothesis. Risk perception influences psychological resilience in part through cognitive resilience, and the magnitude of this relationship depends on the nature of the emergency.

This finding reinforces the idea that psychological resilience is a context-sensitive process, shaped by the interaction between cognitive assessments and the degree of unpredictability of the environment, while emphasizing the importance of calibrating post-disaster psychological interventions according to the specifics of the situation (e.g., stimulating cognitive self-regulation in unpredictable events and training emotional strategies in controllable ones).

As highlighted in Table 5, the obtained moderate mediation index [Index = −0.0433, Boot SE = 0.0100, 95% CI (−0.0651, −0.0254)] is negative and significant, which indicates the presence of genuine moderate mediation, according to the criteria proposed by Hayes (2022).

The results confirm that the magnitude of the indirect effect of risk perception on psychological resilience, mediated by cognitive resilience, varies significantly depending on the nature of the emergency. More specifically, the data suggest that in conditions perceived to be more severe and unpredictable, such as fires, the mediated effect is more pronounced, reflecting more intense cognitive engagement (Bakic and Ajdukovic, 2021) in adaptive regulation processes (Mouguiama-Daouda et al., 2026). On the other hand, in more controllable situations, such as floods, cognitive mediation has a lower intensity, which indicates that psychological adaptation is supported to a greater extent by direct affective and behavioral mechanisms (Wu et al., 2025).

Therefore, the results in Table 5 reinforce the empirical evidence supporting the validity of the hypothetical H2 model and confirm that the interaction between cognitive and contextual factors determines how risk perception influences psychological adaptation. The results support the interpretation that cognitive resilience acts as a dynamic, flexible, and context-dependent mechanism, whose effectiveness varies according to the severity and unpredictability of the threatening situation (Lomeli-Rodriguez et al., 2025).

This result is consistent with recent literature approaching resilience from a contextual and multidimensional perspective (Bonanno, 2021; Southwick and Charney, 2022; Kalisch et al., 2019). According to these approaches, resilience is not a fixed feature, but an adaptive process that involves the complex interaction between cognitive resources, emotional state, and situational threat assessment.

From an applicative perspective, the results highlighted in Table 4 emphasize the importance of differentiating post-disaster psychological interventions according to the specific context. In situations characterized by a high degree of uncertainty and danger, such as fires, interventions should aim to strengthen cognitive control and self-regulation strategies to reduce cognitive overload and facilitate emotional recovery processes. Conversely, in more predictable events, such as floods, interventions can emphasize developing emotional strategies, regaining a sense of control, and restoring emotional balance. Thus, the significant moderate mediation index confirms that psychological resilience does not depend exclusively on the perception of risk, but also on the way in which cognitive mechanisms are activated and calibrated in relation to situational particularities.

Overall, these results offer an integrative perspective on the process of coping with stress and how individuals modulate their cognitive and emotional responses to manage critical situations effectively. Additional results confirm the dynamic, contextual, and multidimensional nature of the resilience process, demonstrating that the impact of risk perception on psychological resilience is not linear, but rather depends on the interaction between cognitive regulatory processes and the situational specificity of the threat.

From an applicative perspective, they provide an empirical basis for the development of differentiated psychological interventions, adapted to the type of disaster, which strengthen cognitive skills in situations of high uncertainty and favor emotional regulation in the most predictable ones.

**Hypothesis 3 (H3)**. Previous experience with natural disasters (floods, fires) predicts the level of individual resilience (psychological, cognitive, adaptive, institutional), so people who have gone through at least one event show different levels of resilience compared to people without similar experiences.

The Kolmogorov–Smirnov and Shapiro–Wilk tests were applied separately to participants who had and those who had not experienced natural disasters.

As shown in Table 6, all four dimensions of resilience—psychological, cognitive, adaptive, and institutional—show significant deviations from the normal distribution (*p* < 0.05), indicating nonparametric distributions of the data.

**Table 6:** Normality tests for resilience dimensions according to previous experience with natural disasters (*N* = 172).

**Variable**	**Experience**	***n***	**Kolmogorov–Smirnov D**	***p***	**Shapiro–Wilk W**	***p***	**Assessment**
Psychological resilience	0 = no experience	20	0.246	0.003	0.855	0.006	Non-normal
	1 = with experience	152	0.143	0.000	0.957	0.000	Non-normal
Cognitive resilience	0 = no experience	20	0.197	0.041	0.892	0.029	Non-normal
	1 = with experience	152	0.084	0.010	0.981	0.032	Non-normal
Adaptive resilience	0 = no experience	20	0.323	0.000	0.761	0.000	Non-normal
	1 = with experience	152	0.150	0.000	0.955	0.000	Non-normal
Institutional resilience	0 = no experience	20	0.194	0.047	0.898	0.037	Non-normal
	1 = with experience	152	0.110	0.000	0.972	0.003	Non-normal

In the inexperienced group, the Shapiro–Wilk test showed significant values for psychological resilience (W = 0.855, *p* = 0.006), cognitive resilience (W = 0.892, *p* = 0.029), adaptive resilience (W = 0.761, *p* = 0.000), and institutional resilience (W = 0.898, *p* = 0.037).

Also, for the experienced group, all four dimensions show significant deviations from normal (all *p* < 0.05).

The results confirm that the assumption of normality is not met; therefore, the analyzed distributions are nonparametric. To test the H3 hypothesis—regarding the differences in the level of resilience according to previous experience with natural disasters (Table 7)—the Mann–Whitney U nonparametric test was used, suitable for comparing two independent groups under the conditions of a nonparametric distribution of data (Field, 2024; Pallant, 2020).

**Table 7:** Results of the Mann–Whitney U test for resilience differences according to previous experience with natural disasters (*N* = 172).

**Resilience dimension**	**Group 0 (no experience, *n* = 20) mean rank**	**Group 1 (experienced, n = 152) mean rank**	**In the**	**With**	***p* (bilateral)**	**Interpret**
Psychological	49.40	91.38	778.00	−3.57	0.000	Significant difference
(Cognitive)	50.20	91.28	794.00	−3.49	0.000	Significant difference
(Adaptive)	28.50	94.13	360.00	−5.58	0.000	Significant difference
(Institutional)	68.40	88.88	1158.00	−1.75	0.081	Insignificant difference

The negative values of the Z statistics indicate that the “experienced” group has higher average ranks. The significance level *p* < 0.05 indicates statistically significant differences.

The interpretation of the results in Table 7 highlights statistically significant differences between people with and without previous experience of natural disasters in three of the four dimensions of resilience.

Analysis by the Mann–Whitney U nonparametric test showed that people who have experienced at least one flood, fire, or other critical event have significantly higher levels of psychological resilience (U = 778.00, Z = −3.57, *p* < 0.001), cognitive resilience (U = 794.00, Z = −3.49, *p* < 0.001), and adaptive resilience (U = 360.00, Z = −5.58, *p* < 0.001) compared to participants without similar experiences.

The negative values of the Z statistics indicate that the average ranks are higher among the experienced group, suggesting a stronger ability to manage stress and adapt to challenging contexts.

For the institutional resilience dimension, the observed difference between groups did not reach the threshold of statistical significance (U = 1158.00, Z = −1.75, *p* = 0.081), although the trend of values indicates a similar direction to that of the other dimensions.

This result suggests that, unlike personal resilience (psychological, cognitive, adaptive), which is directly strengthened by the experience of coping with extreme situations, institutional resilience may depend more on the organizational framework, community support, and external structures involved in disaster management.

Overall, the results confirm the H3 hypothesis and support the idea that previous experience with natural disasters contributes to the development of internal coping resources, cognitive flexibility, and emotional stability.

Participants who have experienced such events appear to exhibit a greater capacity for psychological regulation and a more effective cognitive attitude in the face of stress, which confirms the importance of controlled exposure to complex situations in the development of individual resilience.

**Hypothesis 4 (H4)**. Previous experience with natural disasters (floods, fires) moderates the relationship between risk perception and adaptive resilience, so people with direct experiences more effectively transform risk perception into adaptive behaviors.

Moderation Analysis (Model 1, PROCESS v4.2) (Hayes, 2022) (Table 8) was used to test the hypothesis that previous experience with natural disasters (such as floods and fires) modulates the relationship between risk perception and adaptive resilience.

**Table 8:** Moderation model results (Model 1, PROCESS v4.2) (Hayes, 2022) on the relationship between risk perception and adaptive resilience, moderated by previous experience with natural disasters (*N* = 172).

**Section A—model coefficients**					
**Effect/predictor**	**Coefficient (b)**	**Standard Error (SE)**	**t**	**p**	**95% CI (LLCI, ULCI)**	**Interpret**
Constant	1.8284	1.8996	0.96	0.337	(−1.9219, 5.5786)	Insignificant
Risk perception (X)	0.1066	0.0429	2.48	0.014	(0.0219, 0.1913)	Significant main effect
Previous experience (W)	−1.6120	2.1004	−0.77	0.444	(−5.7586, 2.5346)	Insignificant direct effect
Interaction X × W	0.0774	0.0458	1.69	0.093	(−0.0130, 0.1678)	Significant marginal moderating effect
**Section B—model indicators**		
**Indicators de model**	**Value**	**Interpret**
R = 0.7371	—	High multiple correlation
R^2^ = 0.5433	—	The model explains 54.3% of adaptive resilience variance.
F(3,168) = 66.61, *p <* 0.001	—	The overall pattern is significant.
ΔR^2^ (interaction) = 0.0078, F(1.168) = 2.86, *p =* 0.093	—	The contribution of the interaction term is marginally significant.

CI, confidence interval; b, non-standardized coefficient; SE, standard error; ΔR^2^, the variation further explained by the interaction term. Analysis performed with the PROCESS v4.2 macro (Model 1) (Hayes, 2022).

The results indicate that the overall model is statistically significant, F(3, 168) = 66.61, *p* < 0.001, accounting for 54.3% of the variance in adaptive resilience (R^2^ = 0.5433). The high value of the multiple correlation coefficient (R = 0.7371) indicates that the set of variables included in the model consistently contributes to the prediction of the level of adaptation.

The primary effect of risk perception on adaptive resilience is positive and statistically significant [b = 0.1066, *p* = 0.014, 95% CI (0.0219, 0.1913)], suggesting that as individuals perceive the risk associated with natural disasters more intensely, they exhibit higher levels of psychological and behavioral adaptive capacity. In other words, risk perception serves as a cognitive mechanism for activating adaptive strategies, thereby strengthening the capacity for flexible and solution-oriented responses.

The direct effect of previous experience on resilience is not significant [b = −1.6120, *p* = 0.444, 95% CI (−5.7586, 2.5346)], indicating that mere exposure to extreme events does not automatically cause an increase or decrease in adaptive resilience. However, the interaction term between risk perception and experience [b = 0.0774, *p* = 0.093, 95% CI (−0.0130, 0.1678)] is marginally significant, and ΔR^2^ = 0.0078 shows a modest but theoretically relevant additional contribution of the interaction to the model. This suggests the existence of a moderating trend, according to which the effect of risk perception on adaptive resilience is stronger for people who have had direct experiences with natural disasters, compared to those who have not experienced such events.

In psychological terms, the results indicate that previous experience acts as a factor in amplifying the relationship between risk perception and adaptive behaviors. Individuals who have been exposed to crises seem to transform cognitive risk assessments more effectively into adaptive responses, using previous experiences as learning and adjustment resources. In contrast, people without similar experiences tend to show a weaker relationship between risk perception and resilience, due to the lack of relevant behavioral or emotional landmarks.

The H4 hypothesis is partially confirmed. Previous experience with natural disasters does not have a direct effect on adaptive resilience but influences the direction and intensity of the relationship between risk perception and psychological adaptation. This finding supports theoretical models of experiential resilience (Bonanno, 2021; Norris et al., 2008) according to which exposure to stressful events can strengthen cognitive and behavioral coping mechanisms when accompanied by a realistic and constructive perception of risk.

**Hypothesis 5 (H5)**. Previous experience with natural disasters is positively associated with psychological resilience through cognitive resilience, indicating a mediating role of cognitive resilience in this relationship.

Simple Mediation Analysis (Model 4, PROCESS v4.2) (Hayes, 2022) (Table 9) was used to examine whether cognitive resilience explains the mechanism by which previous experience with natural disasters influences psychological resilience. The overall model is significant, F(2, 169) = 41.05, *p* < 0.001, accounting for 33.7% of the variance in psychological resilience (R^2^ = 0.3375). This value indicates a moderate-to-high association between prior experience and cognitive and psychological resilience.

**Table 9:** Results of the mediation model (Model 4, PROCESS v4.2) (Hayes, 2022) on the relationship between previous experience with natural disasters and psychological resilience, mediated by cognitive resilience (*N* = 172).

**Section A—ways of the mediation model**					
**Effect/path**	**Coefficient (b)**	**Standard error (SE)**	***t***	***p***	**95% CI (LLCI, ULCI)**	**Interpret**
Path a: experience → cognitive resilience	2.3184	0.6511	3.56	<0.001	(1.0331, 3.6037)	Significant effect
Path b: cognitive resilience → psychological resilience	0.6940	0.0856	8.11	<0.001	(0.5251, 0.8630)	Significant effect
Path c′: experience → psychological resilience (direct)	1.2962	0.5483	2.36	0.019	(0.2138, 2.3785)	Significant direct effect
**Effect indirect (a** **×b)**	1.6091	0.5031	–	–	(0.6708, 2.6554)	significant mediation effect
**Section B—model indicators**		
**Indicator**		**Value interpret**
R =0.2708	–	A modest relationship between experience and mediator.
R^2^ =0.0734	–	experience explains 7.3% of the variance in cognitive resilience.
R =0.5810	–	moderate-high correlation between set X, M, and Y.
R^2^ =0.3375	–	The complete model explains 33.7% of the variance in psychological resilience.
F(2,169) = 41.05, *p <* 0.001	–	Significant overall pattern.
Indirect effect (Bootstrap 5,000) = 1.6091, 95% CI (0.6708, 2.6554)	–	Mediation effect confirmed.

CI, confidence interval; b, non-standardized coefficient; SE, standard error; ΔR^2^, the variation further explained by the mediator. Indirect effects are estimated based on 5,000 bootstrap samples. Analysis performed with the PROCESS v4.2 macro (Model 4; (Hayes, 2022).

The results show that previous experience with extreme events (floods, fires) has a positive and significant effect on cognitive resilience [b = 2.32, *p* < 0.001, 95% CI (1.03, 3.60)]. This suggests that people who have experienced natural disasters develop more intense cognitive coping mechanisms, such as flexible thinking, positive reappraisal, and solution-oriented planning. The pathway between cognitive resilience and psychological resilience is also significant and positive [b = 0.69, *p* < 0.001, 95% CI (0.53, 0.86)], confirming that adaptive cognitive functioning supports emotional balance and the ability to self-regulate in the face of stress.

The direct effect of experience on psychological resilience remains significant [b = 1.30, *p* = 0.019, 95% CI (0.21, 2.38)], indicating that experience has an independent impact on emotional balance, but to a lesser extent than the total effect. The indirect effect (a × b) of experience on psychological resilience, via cognitive resilience, is significant 9b = 1.61, BootSE = 0.50, 95% CI (0.67, 2.66)]. Since the bootstrap confidence interval does not include a value of zero, a mediation effect is confirmed. Thus, previous experience influences psychological resilience partly by mediating cognitive resilience, which indicates partial mediation.

These results confirm the H5 hypothesis and support the theoretical model, which posits that difficult experiences can serve as factors for learning and strengthening cognitive coping strategies. Cognitive resilience serves as an explanatory mechanism between experience and psychological balance, meaning that people exposed to traumatic events not only develop emotional reactions of resistance but also cognitive structures that facilitate the reinterpretation of stressful situations and maintain a sense of internal control.

From a theoretical perspective, these results support the processual models of resilience (Bonanno, 2021; Norris et al., 2008; Tugade and Fredrickson, 2004). According to this, psychological adaptation is a dynamic process in which direct experience activates cognitive resources that modulate the emotional response. In this context, cognitive resilience acts as an “adaptive filter”, transforming the impact of negative experiences into opportunities for personal growth and strengthening emotional balance.

Previous experience with natural disasters contributes to psychological resilience, primarily through the development and activation of cognitive mechanisms. The results underscore the importance of incorporating cognitive dimensions into post-disaster intervention programs to facilitate adaptive reinterpretation processes and promote long-term mental health.

## 4 Discussion

This result supports contemporary contextual and processual models of resilience, according to which cognitive adaptation processes are more active and demanding under conditions of acute stress, influencing emotional balance and overall psychological performance (Bonanno, 2021; Kalisch et al., 2019; Chen et al., 2024). In addition, in the broader literature on traumatology, psychological interventions such as cognitive behavioral therapy/acceptance and commitment therapy, or “resilience enhancement” programs are conceptualized precisely as ways to strengthen those cognitive mechanisms that mediate the stressor-psychological adjustment relationship (Mao et al., 2025).

Confirmation of the mediated effect of cognitive resilience on the relationship between risk perception and psychological resilience (H2) suggests that individuals do not react passively to external threats, but instead activate complex cognitive resources—such as planning, positive reassessment, and anticipation—to transform stress into an opportunity for adaptation. However, the adverse effect of cognitive resilience on the psychological component suggests the existence of a compensatory mechanism: intense cognitive activation can temporarily reduce the available emotional resources, a phenomenon explained by psycho-neurocognitive models of overload (Chen et al., 2024; Feder et al., 2019). This result highlights the importance of striking a balance between cognitive control and emotional regulation in stress adaptation processes. This tension between cognitive control and emotional regulation has been explored in intervention research, where some protocols propose alternating between cognitive intervention sessions and emotional support sessions – see the “resilience enhancement” program in Mao et al. (2025).

The finding that the type of hazard moderates the relationship between risk perception and psychological resilience, with more pronounced effects in the case of fires, perceived as unpredictable and dangerous, supports the hypothesis that the intensity and nature of the event conditions the degree of cognitive activation (Paton, 2019; Del Giudice et al., 2011, 2018). Situations perceived as severe require more elaborate cognitive processes, which confirms the theories of adaptive calibration and contextual resilience (Southwick and Charney, 2022; Brooks et al., 2019; Crane et al., 2022; Morganstein and Ursano, 2020). These results complement his research (Tugade and Fredrickson, 2004) on the role of positive emotions and cognitive reassessment in restoring psychological homeostasis after traumatic experiences. In addition, the extensive literature on trauma (including modern theories) argues that contextual factors and situational variability are essential in shaping adaptive response (see recent reviews of psychological interventions after disasters, such as Li et al. (2024). Similarly, the RESILIENT program, applied as a blended intervention for Fort McMurray fire survivors, was evaluated through an RCT and showed that the intervention can support cognitive coping and reduce symptoms of traumatic stress in a context of unpredictable hazard (Békés et al., 2022).

Additionally, the analysis of differences between individuals with and without previous disaster experience (H3) revealed higher levels of psychological, cognitive, and adaptive resilience among those who had experienced critical events. This result supports the theory of experiential learning and resilience building through repeated exposure to stressful situations (Bano et al., 2025; Partelow, 2021; Wu et al., 2025). However, the lack of significant differences in the institutional dimension suggests that systemic resilience depends more on community infrastructures and public policies, not just on individual experiences (Norris et al., 2008; Ungar, 2021). This finding confirms the importance of constructive collaboration between individual and organizational factors in post-disaster reconstruction.

The results related to the H4 hypothesis showed that previous experience marginally moderates the relationship between risk perception and adaptive resilience, indicating a trend in which people previously exposed to disasters are more effective at transforming risk awareness into adaptive behaviors. This finding is consistent with recent literature describing resilience as an experience-based learning and recalibration process (Brooks et al., 2019; Feder et al., 2019; Broche-Pérez et al., 2025; Chen et al., 2023; Xu et al., 2025; Mao et al., 2025; Grover et al., 2025). In practical terms, this result underscores the importance of experiential training and simulations in enhancing the capacity for psychological reaction and adaptation.

In addition to the conditional process analyses, a simple mediation analysis was conducted to examine the explanatory role of cognitive resilience in the relationship between prior disaster experience and psychological resilience.

The confirmation of the H5 hypothesis—which posits that the relationship between previous experience and psychological resilience is mediated by cognitive resilience—underscores the transformative role of flexible thinking and positive reappraisal. Cognitive resilience acts as an adaptive filter, allowing for the reinterpretation of negative experiences, the reduction of perceived threat, and the restoration of a sense of internal control (Tugade and Fredrickson, 2004; Tedeschi et al., 2018). It supports the model proposed by Bonanno (2021) according to which effective adaptation to stress involves a balance between realistic cognitive assessment and the restoration of existential meaning. In this regard, interventions aimed at cultivating cognitive flexibility (such as CBT (Cognitive Behavioral Therapy)/ACT (Acceptance and Commitment Therapy) therapies, the training program for disaster interventionists) are relevant: for example, (Mahaffey et al., 2021) evaluated a Disaster Worker Resiliency Training, which includes cognitive components of adaptation and reframing, and found beneficial effects on reducing stress symptoms in intervention personnel. Also, interventions that combine mindfulness, self-control, and cognitive strategies are recommended in the MHPSS (Mental Health and Psychosocial Support) literature after seizures (Li et al., 2024).

Overall, the results of this study validate a processual and multidimensional model of resilience, in which cognitive, emotional, and experiential components interact dynamically. From a theoretical perspective, these data confirm the relevance of moderate mediation models (Hayes, 2022) for the study of psychological adaptation, providing an integrative perspective on the relationship between risk perception and post-disaster resilience. From an applicative perspective, they emphasize the need to calibrate psychological interventions according to the type of event and the cognitive profile of the affected individuals. Interventions aimed at strengthening cognitive self-regulation can be essential in situations with a high degree of uncertainty (e.g., fires), while strategies based on emotional support and restoring meaning are more effective in predictable contexts (e.g., floods).

In conclusion, the research demonstrates that psychological resilience is the result of the complex interaction between subjective perceptions of risk, cognitive mechanisms of regulation, and previous experience with natural disasters. This perspective provides a solid basis for the development of differentiated prevention and intervention programs, which include cognitive, emotional, and experiential components, thus contributing to increasing the capacity for individual and community adaptation in the face of emerging climate risks (Intergovernmental Panel on Climate Change, 2023).

## 5 Conclusions

The results obtained in the study confirm that psychological resilience is a complex, dynamic, and interdependent process, simultaneously influenced by risk perception, cognitive regulatory mechanisms, and previous experiences with natural disasters. The analyses carried out highlighted that the perception of risk exerts a significant effect on psychological resilience, and this relationship is mediated by cognitive resilience, which functions as a mechanism for transforming stress into adaptation. People who were able to mobilize cognitive resources—such as positive reappraisal, anticipation, and mental flexibility—showed a greater capacity to restore emotional and rebuild psychological balance after exposure to critical events.

The results also confirm the role of previous experience as a moderating factor in the adaptive process. Individuals who have previously experienced natural disasters exhibited higher levels of psychological and cognitive resilience, indicating that controlled exposure to stressful situations may foster adaptive learning and enhance self-regulation skills. At the same time, it has been observed that the intensity and nature of the event influence the coping mechanisms. Disasters perceived as unpredictable or violent result in a more intense cognitive and emotional activation, as well as a slower reconstruction of the affective balance.

The comparative analysis of the dimensions of resilience revealed that the differences between individuals with and without previous experience are more pronounced in the psychological and cognitive components, while institutional resilience remains constant. This indicates that individual coping mechanisms can be strengthened through experience and education, but community reconstruction processes depend to a greater extent on organizational and infrastructural support.

From a theoretical perspective, the study contributes to clarifying the relationships between risk perception, cognitive and psychological resilience, proposing an integrative explanatory model based on the interdependence among cognitive assessments, emotional regulation, and experiential learning. This processual perspective offers a more nuanced understanding of how individuals transform stress into an adaptive resource and the role of experience in fostering resilience.

From an applicative perspective, the conclusions obtained emphasize the importance of differentiating post-disaster psychological interventions according to the specifics of the event and the cognitive profile of the affected people. In situations characterized by unpredictability and acute danger, emphasis should be placed on training cognitive flexibility, mind control techniques, and stress management. In more predictable contexts, restoring emotional coherence, seeking social support, and regaining a sense of security become priorities.

Overall, the research demonstrates that psychological resilience is not just an individual trait, but a dynamic process of adaptation, involving the simultaneous restructuring of cognitions, emotions, and behaviors in relation to life experiences. The study's contribution lies in the empirical validation of a processual explanatory model of resilience, which can guide both personalized psychological interventions and psychological preparedness and recovery policies in the face of natural disasters.

## 6 Limitations of the study

Although the present research makes a significant contribution to understanding the processual mechanisms of psychological resilience in natural disaster contexts, certain methodological and conceptual limitations are worth discussing to guide the interpretation and generalization of the results. First, the cross-sectional design of the study, although adequate for identifying predictive relationships between variables, does not allow causal inferences on the dynamics of mediation and moderation processes. In this context, the moderated mediation analyses should be interpreted as conditional associations rather than as evidence of temporal or causal dependency between the examined mechanisms.

However, given the exploratory purpose and the proposed complex model, this approach provides a solid basis for future longitudinal investigations to test the temporal stability of the identified relationships (Hayes, 2022).

Secondly, the sample used, consisting of 172 teachers, reflects the socio-cultural and professional particularities of the Romanian educational environment. Although this homogeneity ensures the control of potentially confusing cognitive and educational variables, it can limit the generalization of results to other professional categories or cultural contexts. However, the selection of this sample is justified by the specific interest in the cognitive and reflective role of the teaching profession in the process of psychological adaptation, thus reinforcing the contextual validity of the conclusions (Bonanno, 2021; Kalisch et al., 2019; Chen et al., 2024).

Another limitation is the exclusive use of self-reporting methods, which are susceptible to biases related to social desirability or subjective self-evaluation. However, the use of validated psychometric instruments with a high internal consistency (α = 0.79–0.89), confirmed by exploratory factor analysis and adequacy tests (KMO, Bartlett), provides confidence in the accuracy and stability of the measured constructs (Costello and Osborne, 2005; Hair et al., 2022).

Also, important variables such as social support, coping styles, or intensity of perceived stress were not included in the current model. This methodological decision was deliberate, aiming at clearly delimiting the relationships between the fundamental cognitive and experiential variables of resilience. Consequently, the study successfully captured the essence of cognitive processes and provided a clear picture of how they mediate psychological adaptation in crisis contexts.

Finally, the socio-cultural context specific to Romania can influence the way in which risk perception and adaptation mechanisms are internalized and expressed. However, the comparative inclusion of two types of disasters (fires and floods) allowed the validation of the theoretical model in a varied and ecological framework, reinforcing the practical relevance of the results.

Overall, the identified limitations do not diminish the explanatory value of the research but provide fertile directions for further expansion of the investigations, confirming the methodological robustness and theoretical consistency of the proposed model.

## 7 Future research directions

The further development of this field requires a dynamic and multidimensional approach, which captures how cognitive, emotional, and contextual mechanisms interact in the formation of psychological resilience. Future research directions should explore the dynamics of cognitive and emotional resilience longitudinally, as well as the effects of cultural differences on post-disaster psychological coping mechanisms. Longitudinal studies could provide a deeper understanding of the stability of mediation and moderation processes over time, helping to identify the stages of transformation of stress into an adaptive resource (Bonanno, 2021; Chen et al., 2024). Methodologically, future research should include relevant additional variables, such as social support, coping strategies, personality traits, and perception of internal control, to develop integrated explanatory models. In addition, the use of mixed methods (quantitative and qualitative)—through narrative interviews, focus groups, and content analyses—would allow the exploration of subjective meanings and symbolic mechanisms through which individuals give meaning to traumatic experiences (Tedeschi et al., 2018).

From an applicative perspective, future studies can investigate the effectiveness of differentiated psychological interventions according to the type of disaster. Programs based on cognitive training, emotional regulation, and positive restructuring can be empirically tested to assess the impact on post-event recovery and prevention of secondary traumatic stress (Southwick and Charney, 2022). Such a direction would contribute to the scientific substantiation of policies related to psychological training and community reconstruction.

Intercultural comparative research can also clarify how socio-economic factors, collective values, and community norms modulate the adaptation process. The integration of cross-cultural perspectives would allow testing the cross-cultural validity of the proposed model, contributing to the generalization of theories of resilience in different social and climatic contexts (Intergovernmental Panel on Climate Change, 2023; Ungar, 2021).

In conclusion, future research directions should aim not only to expand the processual model of resilience empirically but also to develop evidence-based, applicable interventions that support cognitive and emotional adaptation in the face of emerging ecological and climate threats. Thus, the present research provides a solid foundation for constructing an integrated paradigm of psychological resilience, which combines scientific analysis, cognitive reflection, and social responsibility.

## Data Availability

The datasets presented in this article are not readily available because no datasets of the types listed above were generated or analyzed in this study; therefore, no data restrictions apply. Requests to access the datasets should be directed to MC, mariana.calin@365.univ-ovidius.ro.
